# Rejuvenating the immune system

**DOI:** 10.1002/1878-0261.13802

**Published:** 2025-01-13

**Authors:** Konstantinos Evangelou, Vassilis G. Gorgoulis

**Affiliations:** ^1^ Molecular Carcinogenesis Group, Department of Histology and Embryology, Medical School National and Kapodistrian University of Athens Greece; ^2^ Ninewells Hospital and Medical School University of Dundee UK; ^3^ Biomedical Research Foundation Academy of Athens Greece; ^4^ Faculty Institute for Cancer Sciences, Manchester Academic Health Sciences Centre University of Manchester UK

**Keywords:** aging, immune cells, RANKL, rejuvenation, thymus

## Abstract

Rejuvenation of elementary immune system components has emerged as a promising strategy to deal with increased susceptibility to infections, cancers, autoimmune disorders, and low efficacy to vaccines, frequently accompanying aging. In this context, the thymus has gained significant attention. A recent study by Santamaria et al. reveals that the receptor activator of nuclear factor‐κB (RANK)–RANK ligand (RANKL) axis is altered during age related thymic involution, compromising immune responses. Based on their findings, authors propose exogenous RANKL administration as a therapeutic strategy to reinvigorate thymic function and improve T‐cell immunity during aging.

## Graphical abstract

Rejuvenation of the immune system has gained significant attention to alleviate age‐related disorders and to slow down aging. Strategies toward this direction either focus on lymphatic organs such as the thymus or follow systematic trajectories. Among the latter, recently emerging ones preventing, reversing or even removing immune senescence by exploiting innovative platforms, pave the way for effective future interventions.

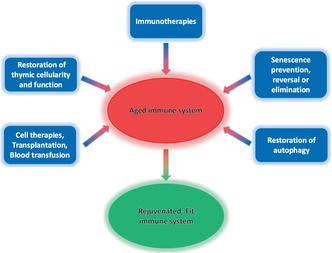

**Abbreviations**
Aire: transcription factors autoimmune regulator
BMP4: bone morphogenetic protein 4
CD150 or SLAM: signaling lymphocytic activation molecule
CD62p: p‐selectin
ECs: endothelial cells
ETPs: early T‐cell progenitors
Fezf2: Fez family zinc finger 2
FOXN1: forkhead box N1
H3K27me3: histone H3 trimethylation at the 27th lysine residue
H3K4me3: histone H3 trimethylation at the 4th lysine residue
H3K9me3: histone H3 trimethylation at the 9th lysine residue
H4K16ac: histone H4 acetylation at the 16th lysine residue
HP1a: heterochromatin protein 1a
HSCs: hematopoietic stem cells
ICAM‐1: intercellular adhesion molecule 1
IGF‐1: insulin growth factor‐1
IL‐7: interleukin‐7
LTis: lymphoid tissue inducer cells
NEO1: neogenin
RANK: receptor activator of NF‐κB
RANKL: receptor activator of NF‐κB ligand
SPs: single positive cells
TCR: T‐cell receptor
TECs: thymic epithelial cells
TGFβRII: transforming growth factor–βRII
TNF: tumor necrosis factor
Tregs: regulatory T cells
VCAM‐1: vascular cell adhesion molecule 1

The immune system with its branches, the innate and the adaptive immune system, is a tightly orchestrated cellular network, coordinated by a wide range of signaling proteins and their receptors, termed cytokines. Given that these molecules influence the immune system spatiotemporally to ensure homeostasis, their deregulation in the context of aging leads to increased infection susceptibility, cancers, and autoimmune disorders. Thus, targeting or restoration of nonphysiological cytokine levels to reinvigorate immune system components has emerged as an attractive direction to alleviate aging and various age‐related processes. Strategies to rejuvenate thymic function and improve T‐cell immunity during aging comprise such a paradigm.

In this context, a recent study by Santamaria et al. [1] elucidates the role of receptor activator of the RANK–RANKL axis in the thymus during aging. The study demonstrates that decreased RANKL levels occur in thymocytes (such as γδ T‐cells, lymphoid tissue inducer cells [LTis], early T‐cell progenitors [ETPs], and CD4+ single positive cells [SPs] during aging), leading to impaired cellularity and function of thymic epithelial cells (TECs) and endothelial cells (ECs), and subsequently to thymic involution. Their findings were recapitulated in young mice by neutralizing RANKL levels, while exogenous RANKL administration in aged mice restored thymic architecture, TEC and EC abundance, and their functional properties. Similarly, RANKL stimulated cellularity and maturation of epithelial and endothelial cells in human thymic organocultures. Moreover, RANKL treatment in aged mice improved T‐cell progenitor homing to the thymus and boosted T‐cell production. As an outcome, peripheral T‐cell renewal and effective antitumor and vaccine responses were achieved.

A critical issue in the study by Santamaria et al. [1] regards the mechanistic basis of age‐related decrease in RANKL levels. Apart from reduced key thymocyte subsets, diminished RANKL expression in these cells was also evident. The aging process is characterized by accumulation of DNA lesions, due to impaired efficiency of DNA repair networks with age as well as by epigenetic changes. The latter include loss of histones (due to telomere shortening and DNA damage, miRNA‐mediated alterations in histone transcription, and impaired balance of activating and repressive histone post‐translational modifications), acetylation, and methylation of DNA or histones (increased histone H4K16ac or H3K4me3 and decreased H3K9me3 or H3K27me3), alterations in levels or activity of chromatin‐associated proteins (increased HP1a), or of noncoding RNAs (such as derepression of transposons) [2]. Overall, they result in profound chromatin modifications reflected by global heterochromatin loss and redistribution [2]. These events subsequently drive transcriptional changes that are anticipated to affect potent RANKL regulators such as hormones, cytokines, and signaling cascade components [3]. As an example, histone demethylases influence lifespan by modulating components of cardinal longevity routes, such as the IGF‐1 axis [2]. IGF‐1 induces RANKL and its levels are known to decrease with age, thus providing a possible explanation of low RANKL expression during thymic involution [2, 3]. In line with this notion, treatment of aged animals with IGF‐1 has been shown to restore thymopoiesis [4]. Another potential mechanism could involve the acquisition of cellular senescence, a hallmark of aging. TCR activation along with TGFβRII stimulation imposes RANKL expression at least in a subset of young thymocytes (CD4^+^ SPs) [5, 6]. T cells reported to be senescent exert impaired TCR signaling that in turn could result in low RANKL levels [7]. Whether these TCR‐deficient T cells are truly senescent is a debatable matter requiring further elucidation [8].

The thymus is a primary lymphoid organ that is vital for the development of T lymphocytes and consequently for adaptive immune responses. T‐cell maturation in the thymus depends on the sequential contribution of phenotypically distinct stromal cell compartments that comprise the thymic microenvironment. Particularly, cortical TECs sustain initial phases of thymopoiesis, involving recruitment of T‐cell progenitors, T‐cell lineage commitment and propagation, and positive selection of CD4+ and CD8+ thymocytes [9]. Medullary TECs in turn orchestrate late thymopoiesis, promoting self‐tolerance by clonal deletion of autoreactive T cells and facilitating the emergence of Tregs [9]. These functions rely on their distinctive property to express a plethora of tissue‐restricted self‐antigens, via Aire and Fezf2 transcription control [9]. Thymic output is also temporally regulated due to age‐related involution, attributed mainly to defects in TEC proliferation and differentiation [1, 10]. In line with this notion, cellular senescence in TEC during thymic involution in humans was reported [11]. Despite these observations, the molecular mechanisms responsible for thymic involution still remain to a large extent elusive.

RANKL is a member of the TNF cytokine family initially identified to play a pivotal role in bone metabolism. Subsequently, it was discovered to be expressed in a wide spectrum of settings including lymphoid tissues, influencing various immune functions. RANKL was detected in activated murine T cells and in bone marrow‐derived stromal cells [12]. Moreover, RANKL was identified as a crucial factor in lymphocyte development and lymph node organogenesis [12]. Regarding the thymus, RANKL was shown to be essential for epithelial cell functions such as proliferation and adhesion of thymocytes to epithelial cells in mice [10]. In the same study, RANKL signaling was accompanied by upregulation of cell death regulatory genes favoring cell survival, cell adhesion molecules such as ICAM‐1 and VCAM‐1, and thymopoietic factors including IL‐7 [10]. RANK‐RANKL axis was shown to regulate the development of medullary Aire‐expressing TECs [10]. Interestingly, RANKL exhibited a significant potential to promote thymic regeneration in mice while RANKL‐deficient mice showed defects in early differentiation of T‐lymphocytes accompanied by the onset of autoimmunity [10].

Among the lymphatic organs, the thymus holds a cardinal position in the implementation of strategies to rejuvenate immune cell populations (Fig. 1). A variety of methods, including exogenous administration of hormones or cytokines, as well as more sophisticated approaches (cell and gene therapy, transplantation) have been proposed toward this direction. In fact, restoration of a single factor, such as the transcription factor FOXN‐1 in TECs or BMP4 in ECs of the involuted thymus, can result in robust thymus regeneration and immune reconstitution [13]. Analogous systematic trajectories exploiting the high plasticity of immune cells have been further adopted (Fig. 1). Some compelling approaches include autophagy reactivation in aged immune cells by the systematic supplementation of spermidine—a polyamine compound involved in metabolic processes—exercise, fasting, intermittent fasting, and long‐term calorie restriction [14]. Another strategy supports the use of antibodies against factors (CD150, CD62p, and NEO1) located on the surface of the hematopoietic stem cell subtype that favors the production of myeloid cells over lymphoid ones in the elderly people, thus facilitating their selective elimination and restoration of adaptive immune responses [15]. HSC‐mediated cell therapy, heterochronic lymphoid organ transplantation, and young blood transfusion have also entered the scenery [16]. Lastly, manipulations to prevent or reverse immune senescence, re‐establishing cell fitness, and effectiveness or removal of senescent immune cells with selective senolytics to alleviate inflammaging (senescence‐induced low‐grade chronic inflammation occurring in advanced age) have recently emerged as innovative strategies [8, 16, 17]. Overall, given that inflammaging, considerably influences both organismal aging and the functionality of immunocompetent cells, systemic approaches that have a broader effect seem the most promising direction in the restoration of immune functions toward slowing down aging and ameliorating accompanying abnormalities.

**Fig. 1:**
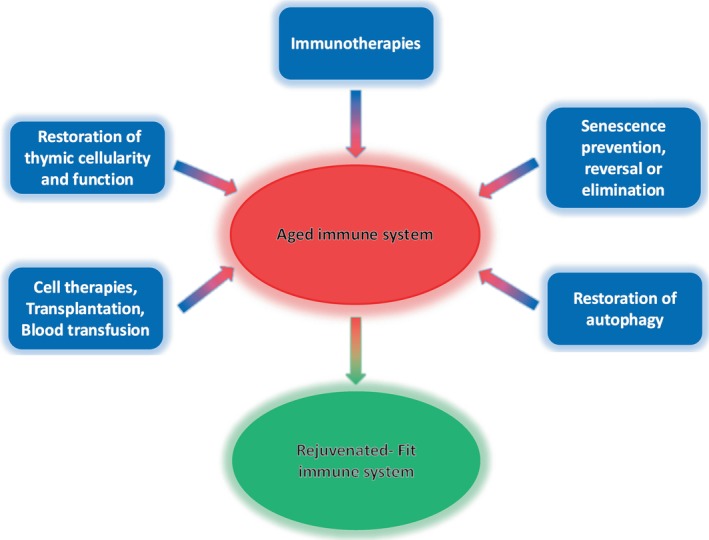
Strategies toward immune system rejuvenation.

## Conflict of interest

The authors declare no conflict of interest.

## Author contributions

KE and VG wrote and edited the commentary.
